# Combination of three miRNA (miR-141, miR-21, and miR-375) as potential diagnostic tool for prostate cancer recognition

**DOI:** 10.1007/s11255-018-1938-2

**Published:** 2018-07-16

**Authors:** Paweł Porzycki, Ewa Ciszkowicz, Małgorzata Semik, Mirosław Tyrka

**Affiliations:** 1 Department of Urology, Municipal Hospital Rzeszow, 4 Rycerska Street, 35-241 Rzeszow, Poland; 2 Department of Biotechnology and Bioinformatics, Faculty of Chemistry, Rzeszow University of Technology, 6 Powstańców Warszawy Street, 35-503 Rzeszow, Poland

**Keywords:** Prostate cancer, Diagnosis, Biomarkers, MiRNA

## Abstract

**Purpose:**

Prostate cancer (PCa) is a common tumor disease in western countries and a leading cause of cancer-driven mortality in men. Current methods for prostate cancer detection, like prostate-specific antigen screening, lead to significant overtreatment. The purpose of the study was to analyze circulating microRNAs in serum as non-invasive biomarkers in patients with diagnosis of prostate cancer and healthy individuals.

**Methods:**

This preliminary study included a population of 20 patients with mean age of 68.6 years and mean PSA of 21.3 ng/ml. Eight healthy patients were used as control. MiRNAs were quantified in the total RNA fraction extracted from serum and levels of five microRNAs (miR-106b, miR-141, miR-21, mir-34a, and miR-375) were quantified by RT-qPCR. Statistical analyses evaluated correlation between clinicopathological data and miRNAs expression levels.

**Results:**

Relative expression ratios of miR-106b, miR-141-3p, miR-21, and miR-375 were significantly increased (1.8-,  1.9-, 2.4-, and 2.6-fold, respectively) in the PCa group compared to healthy control. Using receiver operating characteristics, the highest area under the curve equal to 0.906 was obtained for miR-357 and indicates a very good diagnostic properties of this biomarker. We found expression level of mir-34a not related with PCa.

**Conclusions:**

Our results support previous findings on the possibility of discriminating prostate cancer patients from healthy controls by detecting miRNA (miR-141-3p, miR-21, and miR-375). Further insights into miRNA abundance and characteristics are necessary to validate the panel of miRNA as surrogate markers in diagnosis of prostate cancer.

## Introduction

Prostate cancer (PCa) is a heterogeneous and one of the oncological diseases with a higher incidence in western countries. It includes several phenotypes, from indolent to highly aggressive forms, despite the fact that it tends to grow more slowly than other solid cancers [1]. Circulating prostate-specific antigen (PSA) is currently the most common non-invasive biomarker used to detect PCa, despite the controversies around its use as a screening tool. Actually, the blood levels of PSA are often elevated in men with benign conditions (e.g., prostatitis, urinary tract infection, or benign prostatic hyperplasia). PSA test is of low specificity, because it is specific for the prostate gland, not for PCa [2]. The screening based on PSA blood levels results in a higher incidence of low-risk PCa, most of which require no treatment (active surveillance has been proposed for those patients). Therefore, a search for new, improved biomarkers is necessary for the diagnosis of PCa.

MicroRNAs (miRNAs) are small (18–22 nucleotides) non-coding RNAs that control gene expression posttranscriptionally and are part of the giant non-coding genome [3]. They can modulate the expression of approximately 30–50% of the protein-coding genes in humans and have regulatory function on molecular signaling pathways in the cell. The first studies on PCa-altered miRNAs began in 2007–2008 with the purpose to identify miRNA profiles with diagnostic, prognostic, and predictive abilities [4, 5]. Recently, several miRNA expression profiles have been reported in prostate tumors and they all share an extensive overall deregulation of miRNAs. Moreover, it was demonstrated that miRNA differential expression profiles in prostate cancer can be firmly correlated with its clinical expression. Finally, accumulating data suggest that microRNAs (miRNAs) are promising potential biomarker and can be used in the detection of PCa [6]. Recently, several miRNA expression profiles have been reported in prostate tumors and they all share an extensive overall deregulation of miRNA [4, 5, 7]. A large number of serum miRNAs have been found to be differently expressed in prostate cancer, among which numerous miRNAs are oncomirs, exhibiting overexpression in PCa and promoting tumorigenesis, negatively regulating many tumor suppressor genes [8, 9]. MiRNAs also function as tumor suppressors, exhibiting downregulation in PCa, which can lead to attenuation of inhibition of tumor initiation and development [10, 11].

MiRNAs have a several properties to be excellent biomarkers for the diagnosis of prostate cancer, such as deregulated expression in PCa, circulation in body fluids (non-invasive way of sampling), usage in cancer monitoring, and prognosis. However, there are technical limitation associated with relatively low abundance, instability, and possible intracellular miRNA contamination [12]. Despite these limitations, alteration in circulating miRNAs reflects dysregulation of cancer immunity, cell growth, proliferation and stromal interaction, mediation, and promotion of cellular apoptosis [12] which makes miRNAs a suitable tool to identify diagnostic and prognostic marker.

The aim of this paper is to identify specific miRNAs in patients for routine diagnosis of PCa. We investigated the expression pattern of five cancer-associated miRNAs, miR-106b, miR-141, miR-21, miR-34a, and miR-375 in the serum of PCa patients and control. We also analyzed correlation between circulating levels of the tested miRNAs and clinicopathological characteristics (PSA, Gleason score and European Association of Urology risk group).

## Materials and methods

### Sample collection and hemolysis assessment

In the period between June and December 2017, 62 patients were selected for our study. 52 patients were referred to our department with suspicion of PCa. Digital rectal examination (DRE) and PSA tests were applied for the early detection of PCa. Three patients had negative biopsy before. Patients were diagnosed by an echo-directed transrectal biopsy along with the taking of 10 cylinders and following the performance of PSA analysis. Out of this population, 20 patients with finally diagnosed PCa (19 positive biopsy, one positive specimen from transurethral resection of the prostate procedure (TUR-P)), were selected for miRNAs analysis, the other 10 cases were healthy. All biopsy procedures were performed by one physician. In PCa group, the mean age was 67.5 years (range 49–79), the mean total PSA 21.3 ng/ml (range 3.8–108 ng/ml), and a median of 7 for Gleason grade. Those patients were classified into subgroups, obtaining 5 (25%), 6 (30%), and 9 (45%) patients with low-, intermediate- and high-risk of PCa, respectively (Table 1).

**Table 1:** Clinical characteristics of the patients

Characteristics	Prostate cancer
Age	
Mean	68.6
Range	56–78
Clinical stage	
cT1c	18
cT2c	1
cT3	1
PSA	
< 2.5	0
2.5–10	9
10	4
> 20	7
Gleason score	
6	10
7	5
8	3
9	2
EAU risk group	
Low-risk	5 (25%)
Intermediate-risk	6 (30%)
High-risk	9 (45%)

Eight control patients with mean age 68.6 years (range 56–78) were also recruited and matched by mean age with the individuals affected by PCa. They were referred to our department for other clinical problems (non-PCa).

The ethics committee of the Clinical Research Ethics Committee of Subcarpathian Region approved this study (Nr 10/B/2017), and written consent was obtained from all patients to provide information and samples for research purposes.

2 ml of peripheral whole blood samples was collected from patients before prostate biopsy into tubes without anticoagulant. Blood samples were stored at room temperature for 30 min to allow complete coagulation. Coagulated samples were then centrifuged at 1500×*g* for 15 min at 4 °C to separate serum. The upper phase containing serum was carefully collected, aliquoted in 1.5 ml RNase-free tubes and frozen immediately at − 80 °C, until further use. To avoid quantifying miRNAs in hemolytic serum samples, spectral analysis was performed with readings from 350 to 620 nm. The absorbance peak at 414 nm was indicative for free hemoglobin, with additional peaks occurring at 541 and 576 nm identifying very high levels of hemolysis. According to Kirschner et al., it was assumed that samples were hemolyzed if the A414 reading were higher than 0.2 [13]. The average absorbance values and standard deviations were calculated from triplicates.

### Total RNA extraction and reverse transcription

Extraction of total miRNA was performed from frozen serum using RiboZol™ RNA Extraction Reagent (AMRESCO, LLC) according to the manufacturer’s instructions. Briefly, 1200 µl of Trizol Reagent was added to 400 µl of serum followed by room temperature incubation for 5 min. Then, 320 µl of chloroform was added, samples were vortexed, and incubated for 10 min at room temperature followed by centrifugation at 12,000×*g* for 15 min at 4 °C. About 80% of the upper aqueous phase containing total RNA was transferred to a new tube and precipitated by adding isopropanol. The samples were enriched with 0.8 µg of carrier RNA (MS2 RNA from MS2-phages, Roche) during the isopropanol precipitation. The pellet was washed with 75% ethanol, air-dried, and resuspended in 50 µl of RNase-free water. RNA concentration was measured using fluorometer Qubit 2.0 (Invitrogen, US), and purity was assessed with spectrophotometer UV–Vis (JASCO V-670). Ratio A260/280 near 1.8 indicated that RNA was free of DNA particles and proteins. Total RNA was divided into 5 µl aliquots and stored at − 80 °C until further use. The reverse transcription (RT) reaction mix contained 10 µM of miRNA universal primer (5′-CAGGTCCAGTTTTTTTTTTTTTTTVN-3′) [14], 1 mM each of dNTPs, 1 mM ATP, 10 µM of reverse primers specific to reference genes (RNU6 and SNORD44) (Table 2), 10× Poly(A) Pol Reaction Buffer, 200 U of M-MuLV Reverse Transcriptase (200 U/µl, EurX), 5 U of Poly (A) polymerase *E. coli* (5000 U/ml, Biolabs), and 10 ng of total RNA. RT reaction was carried out at 42 °C for 60 min and 95 °C for 5 min on Gene Amp® PCR System 9700 (Applied Biosystems, US).

**Table 2:** MiRNAs and reference genes primers sequence used in qPCR analysis for circulating miRNA

Accession number	miRNA/reference gene name	Primer forward (5′–3′)	Primer reverse (5′–3′)	Amplicon		
				Nucleotide position		Size (bp)
				Start	End	
004394^a^	*RNU6*	CGCTTCGGCAGCACATATAC	AGGGGCCATGCTAATCTTCT	329	434	106
002750^a^	*SNORD44*	TGATGATAAGCAAATGCTGACTG	GAGCTAATTAAGACCTTCATGTTCAG	8604	8664	61
0004672^b^	*mir-106b*	CGCACTGTGGGTACTTG	GTCCAGTTTTTTTTTTTTTTTGCAG	52	73	22
0000432^b^	*mir-141-3p*	CGCAGTAACACTGTCTGGT	GTCCAGTTTTTTTTTTTTTTTCCATCT	59	80	
0000076^b^	*mir-21-5p*	GCAGTAGCTTATCAGACTGATG	GGTCCAGTTTTTTTTTTTTTTTCAAC	8	29	
0000255^b^	*mir-34a-5p*	GCAGTGGCAGTGTCTTAG	GGTCCAGTTTTTTTTTTTTTTTACAAC	22	43	
0000728^b^	*mir-375*	AGTTTGTTCGTTCGGCTC	GGTCCAGTTTTTTTTTTTTTTTCAC	40	61	

^a^National Center of Biotechnology Information (NCBI) Reference Sequence Number

^b^miRBase: The MicroRNA Database Accession Number

### miRNA expression profiling

The expression level of the selected genes (RNU6 and SNORD44) and miRNAs (miR-106b, miR-141-3p, miR-21, miR-34a-5p, and miR-375) was determined by qPCR, which was performed using the Eco™ Illumina Real-Time PCR System. Based on the annotated sequences of mature miRNAs (http://www.mirBase.org) and selected reference genes (NCBI), specific forward and reverse primers were designed with the use of Primer-BLAST (Table 2) [15]. PCR reaction mixture included 1 µl of RT product, 1× GoTaq® qPCR Master Mix (Promega, US), 10 µM of each specific reverse, and forward miRNA and reference gene primers (Table 2). The qPCR experiments were performed at 95 °C for 5 min, followed by 45 cycles at 95 °C for 20 s, 60 °C for 60 s, and 95 °C for 15 s. The melting curve analysis was carried out to verify specificity of PCR products. To generate standard curves, qPCR amplification of cDNA and its 10-fold dilutions were performed. PCR amplification efficiencies (E) were calculated for each individual miRNA using the following equation: E = [(10^−1/slope^) − 1] × 100. The relative expression levels were determined taking into account different amplification efficiencies of miRNAs and reference gene with the use of Livak 2^−ΔΔC^_T_ method [16]. Endogenous RNU6 gene was used as an internal control for normalizing data.

### Statistical analysis

Data preprocessing and statistical analyses were performed using GenEx v.6.0.1 (MultiD Analyses AB, Göteborg, Sweden) and STATISTICA v.12 (StatSoft, OK, USA) software. Mann–Whitney *U* test and Spearman rank correlation were used to analyze the data. Receiver operating characteristics (ROC) curves were calculated to determine the potential of miRNAs to discriminate between PCa and healthy controls (HC) samples. To combine biomarkers, logistic regression analysis was applied. Statistically significant differences were set at *p* < 0.05 using two-sided test.

## Results

### Selection of miRNAs and reference genes

Five human microRNAs: miR-106b, miR-141-3p, miR-21, miR-34a-5p, and miR-375 were chosen based on literature [3, 6, 7, 11, 13, 17–20]. These miRNAs were the most frequently analyzed and showed the highest diagnostic potential. Two reference genes RNU6 and SNORD44 were selected as they are frequently used as endogenous controls with equal level of expression between normal and cancer cells. The putative reference gene, SNORD44, showed a 1.2-fold lower expression in tumor serum sample (Mann–Whitney *U* test, *p* = 0.0007) and was therefore excluded. Whereas the second reference gene candidate RNU6 showed no significant expression changes (Mann–Whitney *U* test, *p* = 0.11) and was used to normalize subsequent miRNA analyses.

### Statistical analysis of miRNAs relative expression and correlation analysis

**Fig. 1:**
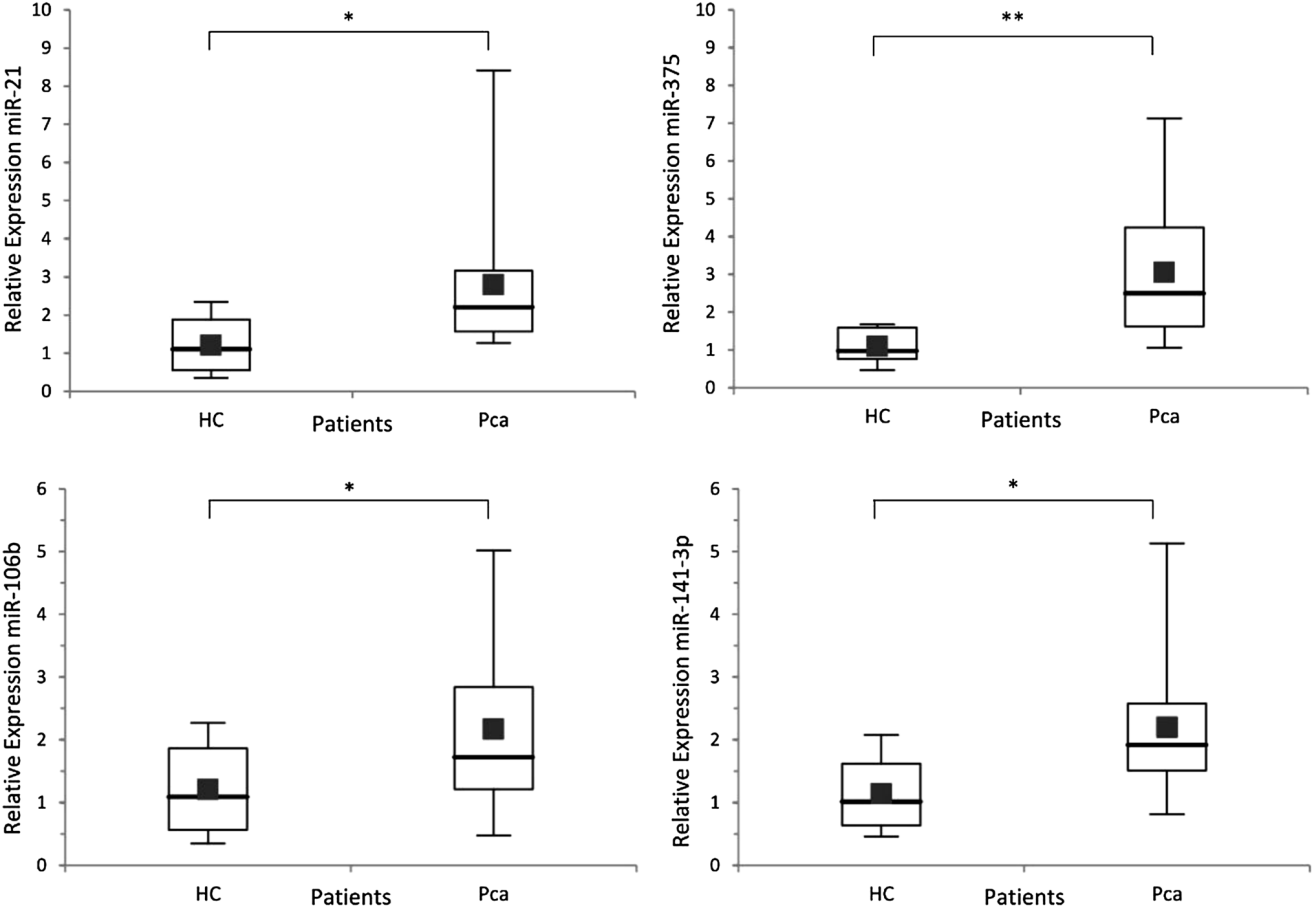
Box plots representing serum miR-21, miR-375, miR-106b, and miR-141-3p expression levels in prostate cancer (PCa) patients and healthy controls (HC). Expression levels of the miRNAs are normalized to RNU6

The expression ratios of selected miRNAs from serum samples of patients with prostate cancer (PCa) and HC were normalized to RNU6. Among them miR-106b, miR-141-3p, miR-21, and miR-375 showed statistically significant differences. No significant difference in the expression of miR-34a-5p were detected between the case and control groups, and this particle was excluded from further analyses. The miR-106b, miR-141-3p, miR-21, and miR-375 showed an increased expression level in the PCa group (expression ratio 1.8, 1.9, 2.4, and 2.6, respectively) (Fig. 1).

Patients enrolled in the study were characterized clinicopathologically (Table 1). The two studied groups did not differ statistically in age, therefore age is not the basis for variability between patients with and without prostate cancer. Whereas, miRNAs expression levels were correlated with PSA value, Gleason score, and European Association of Urology (EAU) risk groups. As the control group consisted of healthy patients who did not have an episode of abnormal prostate function (with no PSA level), the correlation analysis was conducted within the PCa group. Statistically significant interdependence was observed between miR-141-3p and Gleason score (*r*_s_ = 0.655; *p* = 0.015). Expression profiles of the selected miRNA were also compared. Spearman correlation coefficients (*r*_s_) values were evaluated from − 0.323 to 0.655, but statistically significant correlations between miRNAs expression levels were only obtained for miR-141-3p/miR-375 (*r*_s_ = 0.662; *p* = 0.035), miR-141-3p/miR-21 (*r*_s_ = 0.483; *p* = 0.014), and miR-21/miR-375 (*r*_s_ = 0.453; *p* = 0.034).

### MiRNAs as diagnostic markers

**Fig. 2:**
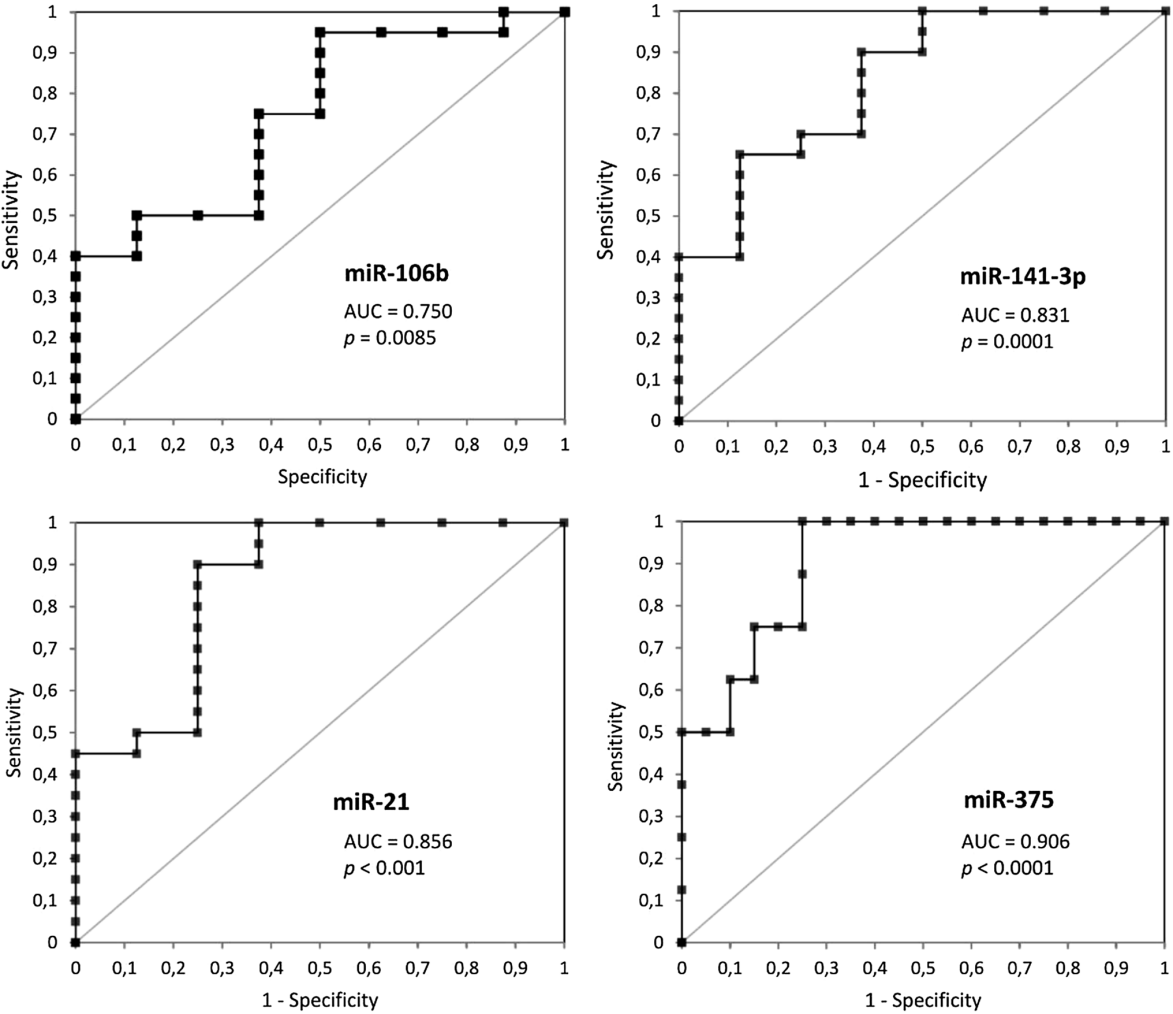
Receiver operating curve (ROC) curve analysis with the use of miR-106b, miR-141-3p, miR-21, and miR-375 to differentiate PCa and healthy controls (HC) samples

ROC analyses were performed to evaluate the capability of miRNAs to discriminate between PCa and non-PCa serum samples (Table 3; Fig. 2). As a result of ROC analysis, the highest AUC was indicated for miR-375 (0.906, 95% CI between 0.797 and 1.001). The miR-21, miR-141-3p showed also a very high AUC values: 0.856 (95% CI 0.680–1.003), 0.831 (95% CI 0.657–1.00), and miR-106b high AUC value equal 0.750 (95% CI 0.545–0.955), respectively.

**Table 3:** The results of ROC analysis for the miRNAs tested and combination

miRNA	AUC	SE	Sensitivity	Specificity
miR-106b	0.750	0.10	0.95	0.50
miR-141-3p	0.831	0.08	0.65^a^ (0.90)^b^	0.88^a^ (0.63)^b^
miR-21	0.856	0.09	0.90	0.75
miR-375	0.906	0.05	1.00	0.75
miR-141-3p/miR-21//miR-375	0.864	0.04	0.93	0.63

AUC values, standard error (SE) sensitivity and specificity are given

^a, b^Sensitivity and specificity with different cut-off values

**Fig. 3:**
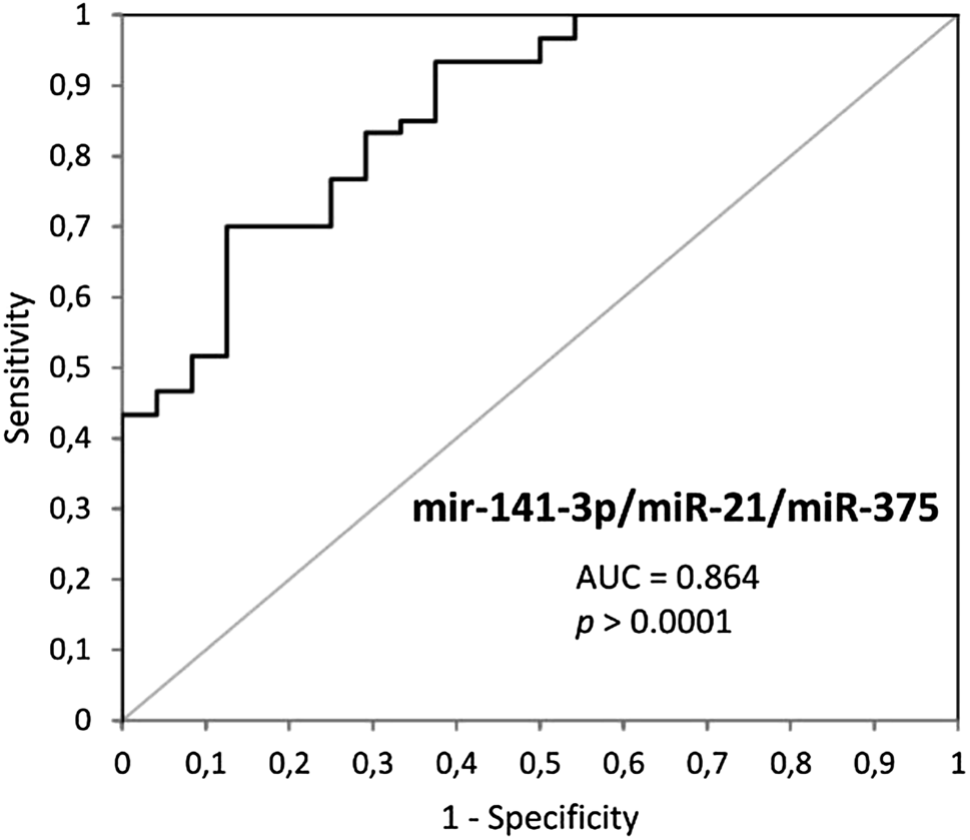
Multimarker ROC curve analysis by combinations of miR-141-3p, miR-21, and miR-375 to differentiate PCa from healthy controls

The cut-off value was chosen to maximize both, sensitivity and specificity, by applying the Youden’s index (Maximum = Sensitivity + Specificity − 1) for each ROC analysis (Table 3). Comparative analysis of diagnostic power of more than one circulating miRNA showed no significant improvement of AUC values for miR-106b, miR-141, and miR-21. However, it was found that combinations of three studied miRNAs (miR-141, miR-21, and miR-375) provide the highest positive predictive value (0.93) with AUC lower than AUC for miR-375 (0.864 and 0.906), respectively (Fig. 3).

## Discussion

In our study, five prostate cancer-specific miRNAs were successfully detected in the serum samples of PCa patients and four of them (miR-106b, miR-141, miR-21, miR-375) showed different abundance when compared with healthy patients. Real-Time qPCR analysis revealed that all four miRNAs were overexpressed in PCa serum samples compared with healthy controls which is in agreement with other studies [21, 22]. Haldrup et al. [21] demonstrated 14.66-fold change in miR-375 expression in PCa serum samples and rather low level of diagnostic properties (AUC = 0.650). Our results differ in values of fold change expression and AUC, indicating a lower differentiation between PCa and HC groups, but higher diagnostic values (expression level 2.6 and AUC = 0.906, respectively). This could be caused by involving different negative controls: benign prostatic hyperplasia (BPH) by Haldrup et al. (2014) and patients with lack of prostate changes arranged in our study. High diagnostic value of mir-141-3p revealed in our study (AUC = 0.831) was also confirmed by Gonzales et al. [22] and Mitchell et al. [23] with AUC comparing PCa and healthy patients equal to 0.907. Brase et al. [12] described miRNA-375 and miRNA-141-3p as the most pronounced markers for high-risk tumors (elevated level in metastatic PCa patients and significant association with poor overall survival) [24]. MiR-375 and miR-141 levels also correlate with high Gleason score [12], which is partially confirmed in our study (correlation between miR-141-3p and Gleason score was significant at *p* = 0.015). Consistent with previous reports [21], levels of miR-141-3p and miR-375 revealed direct correlation (*r*_s_ = 0.662 and *p* = 0.035). Their observations suggest that miRNA-375 and miRNA-141 expression is enhanced in prostate cancer specimens and their release into the blood is further associated with advanced cancer disease, which is partially confirmed in this study. Also miR-21 demonstrated significant correlation with miR-375, confirmed by Gao et al. [25], and miR-141-3p (*r*_s_ = 0.453; *p* = 0.033 and *r*_s_ = 0.483; *p* = 0.014, respectively). The study also concerned the identification of less studied miR-106b, as it appears to be a PCa oncogene, and was suggested as a candidate prognostic marker for prostate cancer [26]. We observed 1.8-fold significant increase of miR-106b expression in PCa group compared with 1.03-fold increase obtained by Sun et al. [27]. Nevertheless, miR-106b showed no significant correlation with clinicopathological data (Gleason score, PSA) or other studied miRNAs and also indicated the lowest significance as diagnostic test (AUC = 0.750; *p* = 0.1).

In our study, we failed to validate miR-34a as prostate cancer biomarker. The expression level of this oligonucleotide was probably too low for reliable testing [28]. miR-34a was downregulated in prostate cancer and inhibited malignant growth by repressing genes involved in various oncogenic signaling pathways [29] and it is suggested that miR-34a therapy may be among the first miRNA mimics to reach the clinic. However, different studies provided opposite data on expression of miR-34a no significant dysregulation in tissue [30] and serum (this study), 0.8-fold downregulation in tissue [31] or upregulation [32].

Three miRNAs miR-141-3p, miR-375, and miR-21 repeatedly appear in various studies and are characterized as being highly overexpressed in PCa, playing important role in tumor growth processes [8, 33, 34]. In order to evaluate the diagnostic value of combination of the three mentioned miRNAs, we performed correlation analyses of expression levels to identify miRNA combinations, which could potentially supplement each other in a diagnostic test, and thus increase sensitivity when compared to individual miRNAs. Testing pair of miRNA resulted in no further improvement of high AUC values observed for single miRNAs (miR-141-3p, miR-21, and miR-375, AUC values were 0.831, 0.856, and 0.906, respectively). However, the combination of three miRNAs (miR-141-3p, miR-21, and miR-375) in a single test would significantly increase predictive value (to 93%), which indicate the probability that the prostate cancer develops when the test is positive, which would be essential for reducing the quantity of overdiagnosis events in prostate cancer treatment.

In conclusions, our results have revealed that miR-106b, miR-141-3p, miR-21, and miR-375 are overexpressed in serum samples of PCa patients when compared with healthy patients. These data are promising for the use of these serum miRNAs for non-invasive and specific detection of PCa. Moreover, our study indicates that combined analysis of serum miR-141-3p, miR-21, and miR-375 can significantly improve the prediction level of the presence of prostate cancer. Within the PCa group, we also found no significant correlation between the miRNA expression and PSA value, which is known to be imperfect prostate cancer indicator. Our results support previous findings on the possibility of detecting miRNA in serum sample for PCa diagnosis and that a combination of detected miRNAs stand out as promising panel markers with a diagnostic potential. However, before microRNAs become a tool for routine diagnostics, additional research is required: (i) fully understand miRNA expression in the human body, (ii) elucidate preanalytical variables that impact miRNA detection, (iii) standardize existing or develop novel miRNA detection methods, and (iv) compare the performance of different miRNA detection platforms.

Within 6 months, 38% of all patients enrolled in our study were diagnosed with prostate cancer. The small sample size can be considered as the limitation of the present study, compared to 46–102 samples tested in related microRNAs projects [11, 21, 25]. However, internal controls corresponding to samples (no hemolysis, no protein, or DNA contamination) and procedures (the performance of isolation, and RT-PCR were checked for particular markers) were maintained to assure high quality of data. Moreover, the statistical analysis showed significant differences of miRNAs expression levels between cohorts.
